# The role of retroelements in Parkinson’s disease development

**DOI:** 10.18699/vjgb-25-32

**Published:** 2025-04

**Authors:** R.N. Mustafin

**Affiliations:** Bashkir State Medical University, Ufa, Russia

**Keywords:** Parkinson’s disease, viruses, microRNA, retroelements, болезнь Паркинсона, вирусы, микроРНК, ретроэлементы

## Abstract

Parkinson’s disease is the second most common neurodegenerative disease characterized by accumulation of alpha-synuclein and Lewy bodies in the brain’s substantia nigra. Genetic studies indicate an association of various SNPs, many of which are located in intergenic and intronic regions, where retrotransposons and non-coding RNA genes derived from them reside, with this disease. Therefore, we hypothesize the influence of SNPs in retroelement genes on Parkinson’s disease development. A susceptibility factor is retrotransposons activation with age, since the disease is associated with aging. We hypothesized that alpha-synuclein accumulates in the brain due to its interaction with transcripts of activated retroelements. As a result of a defective antiviral response and a large number of RNA targets for this protein, its aggregates form Lewy bodies in neurons with inflammation and neurodegeneration development in the substantia nigra. As evidence, data are presented on the role of alpha-synuclein in the antiviral response with binding to RNA viruses, which are characterized by the ability to activate retroelements that have evolved from exogenous viruses integrated into the human genome. Activation of LINE1s in the brain, endogenous retroviruses, and LINE1s in the blood serum of Parkinson’s disease patients was detected. An additional mechanism contributing to the progression of the disease is mitochondrial dysfunction due to insertions of Alu elements into their genomes using LINE1 enzymes. Mechanisms of activated retrotransposons’ influence on microRNAs that evolved from them are described. Analysis of the scientific literature allowed us to identify 35 such microRNAs (miR-1246, -1249, -1271, -1273, -1303, -151, -211, -28, -31, -320b, -320d, -330, -335, - 342, -374a, -374b, -421, -4293, -4317, -450b, -466, -487b, -493, -495, -5095, -520d, -576, -585, -6088, -619, -625, -626, -769, -885, -95) associated with Parkinson’s disease, which may become promising targets for its treatment and diagnosis.

## Abstract (ru)

Болезнь Паркинсона – второе по распространенности нейродегенеративное заболевание, характеризующееся накоплением альфа-синуклеина и телец Леви в черной субстанции головного мозга. Генетические исследования свидетельствуют об ассоциации с болезнью различных SNP, многие из которых расположены в межгенных и интронных областях, где локализованы также ретротранспозоны и произошедшие от них гены некодирующих РНК. В связи с этим сделано предположение о влиянии SNP в генах ретроэлементов на развитие болезни Паркинсона. Фактором предрасположенности является активация ретротранспозонов с возрастом, поскольку заболевание ассоциировано со старением. Предложена гипотеза о том, что альфа-синуклеин накапливается в головном мозге вследствие его взаимодействия с транскриптами активированных ретроэлементов. В результате дефектного противовирусного ответа и большого количества РНК-мишеней для данного белка его агрегаты образуют тельца Леви в нейронах с последующим воспалением черной субстанции и активацией нейродегенеративных процессов. В качестве доказательства приведены данные о роли альфа-синуклеина в противовирусном ответе со связыванием с РНК вирусов, которые характеризуются способностью активировать ретроэлементы, произошедшие в эволюции от встроенных в геном человека экзогенных вирусов. Обнаружены также активированные LINE1-ретроэлементы в головном мозге, эндогенные ретровирусы и LINE1 в сыворотке крови пациентов с болезнью Паркинсона. Дополнительный механизм, способствующий прогрессированию болезни, представляет собой дисфункция митохондрий вследствие инсерций в их геномы Alu-элементов с помощью ферментов LINE1. Описаны механизмы влияния активированных ретротранспозонов на произошедшие от них в эволюции микроРНК. Анализ научной литературы позволил выявить 35 таких микроРНК (miR-1246, -1249, -1271, -1273, -1303, -151, -211, -28, -31, -320b, -320d, -330, -335, -342, -374a, -374b, -421, -4293, -4317, -450b, -466, -487b, -493, -495, -5095, -520d, -576, -585, -6088, -619, -625, -626, -769, -885, -95), ассоциированных с болезнью Паркинсона, которые могут стать перспективными мишенями для ее лечения и диагностики.

## Introduction

List of abbreviations AS – alpha-synuclein GWAS – Genome Wide Association Study HERV – Human Endogenous RetroVirus HIV – Human Immunodeficiency Virus HLA – Human Leukocyte Antigen LINE – Long Interspersed Nuclear Element LTR – Long Terminal Repeat, ncRNA – non-coding RNA ncRNA – non-coding RNA NHEJ – non-homologous end joining ORF – Open Reading Frame PD – Parkinson’s disease RC-LINE1 – retrotransposition-competent LINE1 RdDM – RNA-dependent DNA methylation REs – retroelements SINE – Short Interspersed Nuclear Element siRNA – small interfering RNA SNP – Single Nucleotide Polymorphism SVA – SINE-VNTR-Alu SV-SVA – structurally variable SVA TEs – transposable elements TLR3 – Toll-like receptor 3 WEEV – Western equine encephalitis virus WNV – West Nile virus

Parkinson’s disease (PD) is the second most common neurodegenerative disease after Alzheimer’s disease, affecting 2 % of the world’s population over 65 years of age (Morais et al., 2016). PD is characterized by the degeneration of dopaminergic neurons in the substantia nigra of the brain due to the accumulation of alpha-synuclein (AS) and Lewy bodies in them (Leblanc, Vorberg, 2022). This disease is characterized by prion-like spread of AS (Park et al., 2021). As a result, symptoms such as rigidity, tremors, gait disturbances, and slowness of movement progress clinically slowly. Subsequently, speech, gait, and the performance of daily activities are impaired, and dementia develops (Hossain et al., 2022). The overall heritability of PD risk ranges from 0.27 (Blauwendraat et al., 2019) to 0.36 (Nalls et al., 2019). In most cases, PD is a multifactorial disease associated with polymorphic variants of various genes (Blauwendraat et al., 2019). However, 10 % of patients with PD have monogenic forms of the disease, the most common cause of which are mutations in the LRRK2 gene, which encodes leucine-rich repeat kinase (Oliveira et al., 2021).

A GWAS conducted in 2019 on DNA samples from 28,568 patients with PD identified more than 40 loci reliably associated with PD, including SNPs located in the GBA, INPP5F/SCARB2, LRRK2, MCC1, SNCA, VPS13C genes (Blauwendraat et al., 2019). In another GWAS of the same year, 78 PD-associated polymorphic loci were identified in 37,688 PD patients (Nalls et al., 2019). Most of these SNPs are located in intergenic, promoter and intronic regions (Ohnmacht et al., 2020), where the bulk of retroelement (REs) and non-coding RNA (ncRNA) genes are located (Nurk et al., 2022). Therefore, it can be assumed that the influence of many PD-associated polymorphisms is due to changes in the functioning of REs and ncRNAs, which play a role in regulating the expression of brain neuronal genes (Mustafin, Khusnutdinova, 2020). This is supported by both indirect and direct evidence of the role of REs in the pathogenesis of PD. In particular, the characteristic strong association of PD with aging (only 4 % of PD patients worldwide are under 50 years of age (Hossain et al., 2022)) may be due to the activation of REs during aging (Gorbunova et al., 2021) due to DNA methylation and heterochromatin destruction changes (Ravel- Godreuil et al., 2021).

REs are transposable elements (TEs), which are specific regions of the genome that move to new loci by a “copy and paste” mechanism. TEs also include another class, DNA transposons, which use a “cut and paste” mechanism (Gorbunova et al., 2021). In total, transposons occupy about 1.4 billion bp in the human genome, which is 46.7 % of all DNA sequences. The largest share is made up of autonomous LINEs (0.63 billion bp) that do not contain long terminal repeats (LTR) and non-autonomous SINEs (0.39 billion bp) containing LTR REs (human endogenous retroviruses (HERVs)), which make up 0.27 billion bp (Nurk et al., 2022). About 0.13 % of the human genome is occupied by non-autonomous SVA (SINE-VNTR-Alu) REs in the amount of about 3,000 elements (Fröhlich et al., 2024). DNA transposons occupy 0.108 billion bp (Nurk et al., 2022). REs are important sources of evolutionary emergence of ncRNAs such as microRNAs (Mustafin, Khusnutdinova, 2023). This may explain the results of the analysis of the human genome using specific oligonucleotides complementary to transposons, which showed that RE sequences (not only the REs themselves, but also the regulatory elements derived from them, introns, ncRNA genes and tandem repeats) occupy at least 2/3 of the entire human genome (de Koning et al., 2011).

The close relationship between the functioning of REs and the ncRNAs they generate in regulating gene expression suggests the role of transposons as drivers of epigenetic regulation. Therefore, the failure of evolutionarily programmed species-specific control due to individual RE sequence polymorphisms detected by GWAS (Nalls et al., 2019; Ohnmacht et al., 2020; Bantle et al., 2021) under the influence of aging (Gorbunova et al., 2021) and environmental factors (such as past viral infections (Jang et al., 2009; Batman et al., 2015; Marreiros et al., 2020; Park et al., 2021; Leblanc, Vorberg, 2022)) can cause epigenetic dysregulation in the brain, characterized by the most pronounced TEs activity (Mustafin, Khusnutdinova, 2020). As a result, a neurodegenerative process develops, in which the accumulation of AS and Lewy bodies may reflect a failure in the protective mechanisms of cells against hyperactivated REs, which is due to the role of AS in antiviral processes.

## The role of alpha-synuclein in antiviral defense

REs evolved from exogenous viruses (Mustafin, 2018), which explains one of the modern concepts of aging being caused by hyperactivation of REs (Gorbunova et al., 2021), which stimulate the antiviral interferon response with the development of systemic aseptic inflammation, progressive degeneration of organs and tissues (De Cecco et al., 2019). Therefore, the role of REs in the development of PD may be evidenced by both the influence of viruses on PD and the protective function of AS against viruses. Indeed, according to meta-analyses and systematic reviews of the scientific literature, PD is caused by influenza viruses, Coxsackie, HIV, Japanese encephalitis B, West Nile virus (WNV), St. Louis (Jang et al., 2009), influenza A viruses, herpes viruses and flaviviruses. An increased risk of developing PD after hepatitis B and C infections has been identified (Wang et al., 2020; Leblanc, Vorberg, 2022). Influenza A H1N1 virus has been found to promote proteostasis disruption and AS aggregation (Marreiros et al., 2020). Coxsackie virus B3 induces formation of AC-associated inclusion bodies in neurons acting as PD triggers (Park et al., 2021). Neuroinvasive WNV activates AS expression in neurons (Beatman et al., 2015).

A model was presented in which WNV-induced AS localized to endoplasmic reticulum membranes, modulating virus-induced stress signaling and inhibiting viral replication (Beatman et al., 2015). Experiments with infection of mice with the WEEV (western equine encephalitis virus) revealed protein aggregation in many areas of the brain, including the substantia nigra, with loss of dopaminergic neurons, persistent activation of microglia and astrocytes (Bantle et al., 2021). HIV promotes accumulation of AS in neurons, which explains the development of cognitive and motor disorders in HIV-infected patients, among whom the frequency of SNCA/ alpha-synuclein staining is higher than in healthy people of the same age (Santerre et al., 2021).

AS has many biophysical characteristics of antiviral peptides, binding to virus-carrying vesicles. AS promotes neuronal resistance to viral infections by signaling the immune system and recruiting neutrophils, macrophages, and activating dendritic cells. It has been noted that chronic gastrointestinal infections can lead to the accumulation of AS forming neurotoxic aggregates, as from there AS enters the brain, providing immunity before infection (Barbut et al., 2019).

The mechanism of AS-induced immune responses to RNA viral infections was investigated and it was determined that AS is required for neuronal expression of interferon-stimulated genes. Human AS knockout neurons failed to induce a broad range of interferon-stimulated genes. In the nuclei of interferon-treated human neurons, AS accumulates, with interferon-mediated phosphorylation of STAT2 depending on its expression and localized together with AS after such stimulation. Increased levels of phosphoserine129 alpha-synuclein are expressed in brain tissue from patients with viral (WNV and VEEV) encephalitis (Monogue et al., 2022). A systematic review of the scientific literature in 2024 showed that SARSCoV- 2 induces AS aggregation, promoting the development of PD by stably binding alpha-synuclein to the S1 protein and activating AS as part of the immune response to infection (Iravanpour et al., 2024).

## Direct role of transposable elements in the development of Parkinson’s disease

AS plays a critical physiological role in immune responses and inflammation. Similar to amyloid-beta in Alzheimer’s disease, AS fibrillation represents the brain’s innate immunity against viruses (Vojtechova et al., 2022). Since REs have an evolutionary relationship with viruses (Mustafin, 2018), it can be assumed that mRNA of pathologically activated REs also contributes to the fibrillization of AS. This is evidenced by the results of a study of the abdominal cavity, in which AS is involved in the normal functioning of the immune system, being a mediator of immune responses and inflammation (Alam et al., 2022). Similar to exogenous viruses, degradation and processing products of Res transcripts are stimulators of the interferon response, which contributes to the development of inflammation (Gazquez-Gutierrez et al., 2021). This can be induced not only by LINE1, but also by non-autonomous Alu, which use the enzymes of activated LINE1 for their own transpositions (Elbarbary, Maquat, 2017). As a result, aseptic inflammation characteristic of aging develops (De Cecco et al., 2019), which has been detected in the brain of mice modeled for PD (Ghosh et al., 2016)

**Fig. 1.**
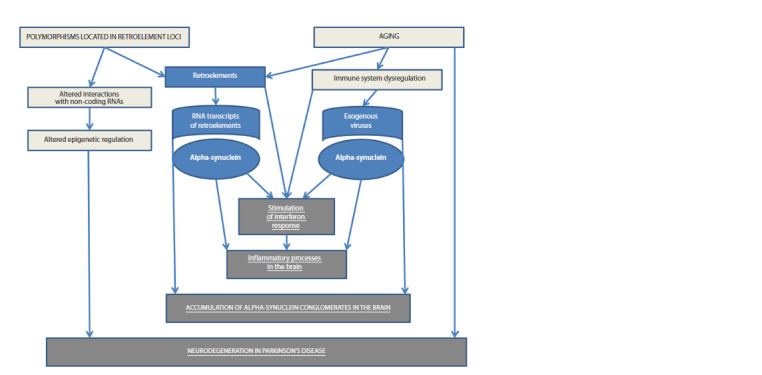
Scheme of retroelements’ involvement in Parkinson’s disease pathogenesis.

In the brain of patients with PD, activation of the immune cytokine network and increased levels of toll-like receptor 3 in response to double-stranded RNA are detected. A C3 complement antisense oligonucleotide, which switches splicing and promotes splicing of unproductive C3 mRNA, has been shown to prevent AS changes (Thomas et al., 2023). The accumulation of pathological AS aggregates (Lewy bodies) in PD may be due to the ineffectiveness of AS action on pathologically activated REs. In the normal brain, REs are also activated, but the interaction of proteins with them may play a role in specific functions of neurons and glial cells. However, in pathological interactions caused by the activation of REs that are not specific to certain structures of the brain (which is due to the spatiotemporal features of REs activation during neuronal differentiation (Mustafin, Khusnutdinova, 2020)), protein conglomerates are formed, especially under the influence of aging (Gorbunova et al., 2021), viruses (Jang et al., 2009; Beatman et al., 2015; Marreiros et al., 2020; Park et al., 2021; Leblanc, Vorberg, 2022) and in the presence of a genetic predisposition caused by polymorphisms in the loci of the location of TEs (Blauwendraat et al., 2019; Nalls et al., 2019; Ohnmacht et al., 2020) (Fig. 1).

Despite the enormous number of REs in the human genome, only a small fraction of them have retained the ability to transpose. This is due to the accumulation of many inactivating mutations during evolution, and the conservation of sequences is due to the use of retroelements by the “hosts” as sources of regulatory elements and ncRNA genes (Mustafin, Khusnutdinova, 2017). For example, LINE1s are distributed in the human genome as over 1 million copies, of which less than 100 have been confirmed to be capable of retrotransposition. Such REs are called “RC-LINE1” (retrotransposition competent LINE1). In addition to these RC-LINE1s, which are contained in the reference genome, there are a small number of non-reference LINE1 insertions (Pfaff et al., 2020).

However, the persistence of activity of even hundreds of REs causes significant insertional polymorphism between individuals, meaning the presence or absence of REs in certain regions of the human genome. Statistical analysis has shown that new Alu insertions occur in every 40th newborn, new LINE1 insertions, in every 63rd, and those of SVA, in every 63rd (Feusier et al., 2019). Whole-genome sequencing showed association of 16 highly active RC-LINE1s with PD compared to healthy controls (Pfaff et al., 2020). 81 reference SVAs were also identified that were polymorphic in presence or absence in PD patients, of which seven were associated with disease progression and PD-specific gene expression changes (Pfaff et al., 2021).

The presence or absence of human-specific SVA_67 correlates with PD progression. SVA_67 exerts a regulatory effect throughout the human genome, being polymorphic in its variable-number tandem repeat (VNTR) domain (Fröhlich et al., 2024). The analysis of polymorphic 2886 Alu, 360 L1, 128 SVA, which are not included in the reference human genome, by their presence or absence in PD compared with healthy controls allowed us to detect REs that have a significant effect on longitudinal changes in clinically significant outcomes of PD (Koks et al., 2022).

LINE1 insertional polymorphisms influence PD progression, as most novel LINE1 insertions are able to regulate gene expression in trans. An association with longitudinal changes in PD progression has been identified for 70 LINE1 markers of degeneration and disease severity (Fröhlich et al., 2023). Using bioinformatics studies and whole-genome sequencing data from 1,000 genomes from different populations, 46 polymorphic HERV-K insertions have been identified. Further analysis of experimental factor ontology enrichment has shown that polymorphic HERV-K insertions (rs12185268, rs17577094, rs17649553, rs183211, rs199515, rs199533, rs415430, rs8070723, rs2395163, rs9275326) are associated with PD features (Wallace et al., 2018).

Non-allelic recombination between homologous repeat elements Alu and LINE1 is widespread in the human genome with tissue-specific features that may act as recombination hotspots. An association between recombination of these REs and genomic instability in PD has been identified (Pascarella et al., 2022). REs are also the cause of most large deletions due to non-homologous end joining (NHEJ) in monogenic forms of PD caused by mutations in the PARK2 gene (Morais et al., 2016). Structurally variable SVAs (SV-SVA) associated with PD and differential gene expression in this disease were identified, which are associated with SNP and differential expression of the BCKDK gene associated with the risk of developing PD. The BCKDK gene encodes branched-chain keto acid dehydrogenase kinase.

The minor risk allele rs14235, located in the BCKDK exon, is associated with a 1.36-fold increase in the mean number of Lewy bodies in PD (Van Bree et al., 2022). Experiments in En+/– mice, a model of PD, revealed loss of heterochromatin and increased LINE1 expression in dopamine neurons. Degeneration of these cells was blocked by direct transcriptional repression using the nucleoside analogue reverse transcriptase inhibitor stavudine, LINE1-targeted small interfering RNAs and expression of viral Piwi1, as well as the specific protein Engrailed, which directly suppresses LINE1 in dopaminergic neurons. LINE1 activation promoted DNA double-strand breaks (Blaudin de Thé et al., 2018). In another study, overexpression of multifactorial protein Gadd45b, involved in DNA demethylation, was induced in the midbrain. In these mechanisms of neurodegeneration, DNA damage was preceded by activated LINE1s with changes characteristic of PD. It has been suggested that aging-related changes in the brain contribute to dopaminergic neurons degeneration with potential implications for PD (Ravel-Godreuil et al., 2021). REs are also sources of DNA damage during aging, which leads to neurodegeneration in PD (Peze-Heidsieck et al., 2022).

The development of PD is also influenced by somatic transpositions in the brain, which affect the biosynthesis of dopamine, serotonin, 3-methoxytyramine, homovanillate, phenethylamine and taurine (Abrusán, 2012). In PD patients, Alu integration into mitochondrial genomes disrupts populations of these organelles in neurons, contributing to the progression of neuronal dysfunction (Larsen et al., 2017). Inhibition of mitochondrial chain complex I in a PD model results in a significant increase in LINE1 element ORF1 protein expression in human dopaminergic LUHMES cells. Activation of these REs was accompanied by loss of DNA cytosine methylation. These mechanisms were blocked by the mitochondrial antioxidant phenothiazine. Such activation of LINE1 is a consequence of mitochondrial distress, which is characteristic of PD (Baeken et al., 2020).

**Fig. 2.**
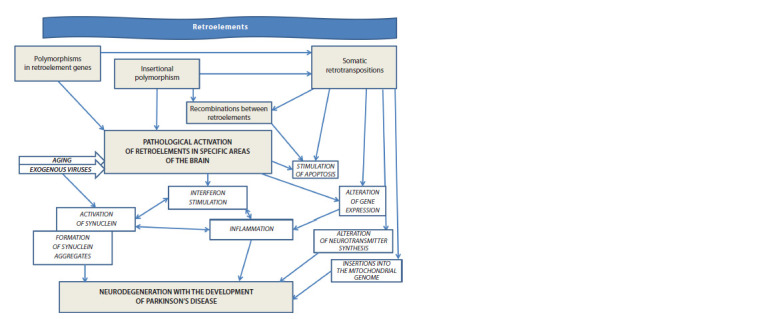
Mechanisms of retroelements’ influence on the development of Parkinson’s disease.

A study of the SVA influence in the composition of the genes of the major histocompatibility complex HLA in patients with PD showed that the expressed alleles of the SVA and HLA genes in circulating leukocytes are differently coordinated in the regulation of immune responses, as well as in the progression of PD (Kulski et al., 2024). Thus, the development of PD can be influenced by structural polymorphisms in the REs genes, the characteristics of the distribution of REs in the genome, reflected in their recombinations and somatic transpositions (Fig. 2).

## Role of retroelement-derived microRNAs in Parkinson’s disease development

An analysis of the scientific literature on changes in the expression of microRNAs originating from REs (according to a published systematic review (Mustafin, Khusnutdinova, 2023)) in PD revealed 35 such microRNAs (see the Table).

**Table 1.**
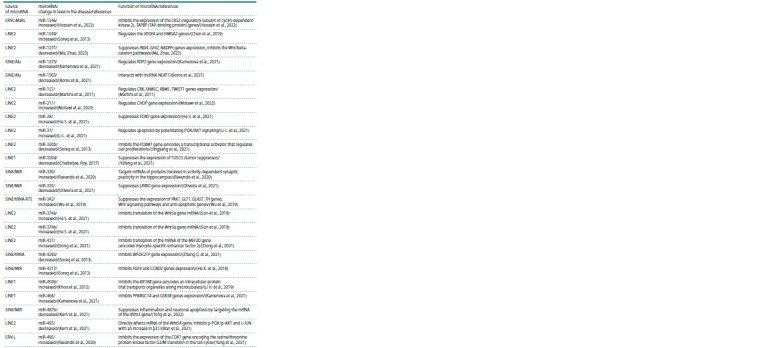
Retroelement-derived microRNAs associated with Parkinson’s disease

**Table 1end.**
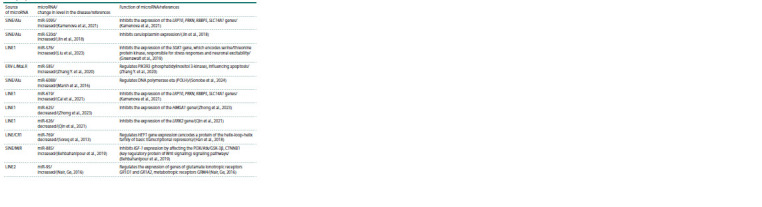
Table 1end.

**Fig. 3.**
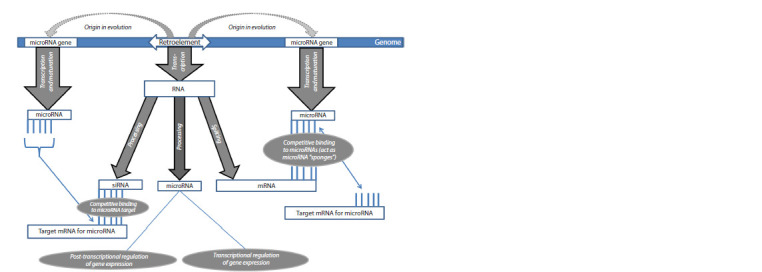
Scheme of the pathways of influence of retroelements on microRNAs derived from them.

Pathological activation of REs in PD may influence the expression of their derived microRNAs in several ways (Fig. 3). First, activated REs act as “sponges” for microRNAs by complementarily binding to nucleotide sequences due to their evolutionary relationship, thus blocking the effects of RNA interference on the mRNAs of the target genes of these microRNAs (Cornec, Poirier, 2023). This regulatory principle has been identified not only in animals but also in plants. For example, the transcript of the LTR-containing retroelement MIKKI (translated from Korean as “bait”), expressed in rice roots, is a mimic for miR-171, which destabilizes the mRNA of root transcription factors like SCARECROW. Processed MIKKI transcripts act as decoys for miR-171, triggering their degradation and promoting the accumulation of root-specific mRNA transcription factors (Cho, Paszkowski, 2017).

Second, LTR-containing REs transcripts (Lu et al., 2014) and LINE1s function as long ncRNA molecules, interacting with specific chromatin regions and regulating the expression of genes controlled by microRNA molecules (Honson, Macfarlan, 2018).

Third, some miRNAs are formed directly from REs genes, which are the basis for pre-miRNA hairpin structures. Such miRNAs lead to spatiotemporal dynamic expression networks, for the analysis of which the Brain miRTExplorer web application was created (Playfoot et al., 2022). Therefore, pathological activation of REs leads to the formation of various microRNAs from their transcripts, which affect the regulatory networks of other microRNAs in the body.

Fourth, REs exert regulatory effects on miRNAs by generating small interfering RNAs (siRNAs) from REs transcripts. In these mechanisms, siRNAs are competitive molecules for binding to mRNA targets of microRNAs, neutralizing their effect on gene expression. This effect is associated with the host cells’ defense systems against activated REs in their genomes, triggering the degradation of REs transcripts by ribonucleases to miRNAs. The latter exert post-transcriptional inhibition of gene mRNAs due to partial complementarity (McCue et al., 2013).

Fifth, one of the ways in which microRNAs interact with REs in regulating gene activity is also the suppression of their expression when microRNAs bind to specific DNA structures formed by REs embedded in these regions.

In the human genome, the Z-form of DNA is formed by endogenous retroviruses, which provide functional genes with alternative promoters (Lee et al., 2022). In addition, the phenomenon of RNA-directed DNA methylation (RdDM) has been described in humans, due to which microRNAs (Playfoot et al., 2022) and miRNAs (McCue et al., 2013) formed from REs transcripts can affect the expression of REs through complementary interactions of sequences in the genome structure (Chalertpet et al., 2019).

## Conclusion

The data presented in the review suggest that the development of PD is caused by the activation of REs as a result of individual characteristics of their distribution and the presence of polymorphisms associated with PD in them. This is evidenced by the following:

1) The results of scientific studies on the association of specific RC-LINE1 sets with PD were obtained. 2) The influence of LINE1 insertional polymorphism on the development of PD was revealed. 3) The significance of 360 LINE1s, 128 SVAs and 2886 Alu in the progression of PD was determined. 4) PD is associated with aging, which is characterized by the activation of REs and the associated inflammation and neurodegeneration. 5) 35 RE-derived microRNAs, the expression of which was significantly altered in PD, were identified. 6) The role of Alu distribution in the genome as a source of mutations in PD was discovered. 7) The influence of Alu insertions into mitochondrial genomes on the progression of PD was determined. 8) The role of synuclein in antiviral protection, with the influence of viruses on the formation of aggregates of this protein, was described.

Similarly, transcripts of pathologically activated REs, evolutionarily related to and interacting with exogenous and viral REs, can stimulate synuclein expression and fibrillization. The probable cause of damage to the substantia nigra is the spatiotemporal features of activation of specific REs in neurons of the brain, which is reflected in the results of their pathological activation in certain most vulnerable areas.

## Conflict of interest

The authors declare no conflict of interest.
